# 

**Published:** 1896-03

**Authors:** 


### CONTENTS:

### SELECTIONS.

Article I.—Dental Science Then and Now. By Dr. S. B. Palmer. 481

" II.—The Origin of Salivary Calculus. By Henry M. Burchard, D. D. S., M. D. - - - - - 497

" III.—A Few Notes on Dental Prosthetics. By. S. Globensky, L. D. S. - - - - - 508

" IV.—Antisepsis. By. J. M. Spear, M. D. - - - 512

### EDITORIAL, ETC.

Systemic Treatment by Dentists. - - - - - - 518

### MONTHLY SUMMARY.

Spitting in Public Places. - - - - - - 520

Infiltration Anesthesia. - - - - - - - 521

To Secure Rubber Dam. - - - - - - - 523

Cleaning Teeth. - - - - - - - - 523

To Raise the Standard of Medical Education. - - - 524

Sterilization of Dental Instruments. - - - - - - 525

Salol Topically. - - - - - - - - 525

A New Substitute for Gold. - - - - - - 526

A Dentist's Surroundings. - - - - - - 526

Salt. - - - - - - - - - - 526

Coal Oil in the Laboratory. - - - - - - 527

Changes in Nerve-Cells. - - - - - - 527

Are Rhubarb Leaves Poisonous? - - - - - 527

Pacific Coast Dental Congress. - - - - - - 527

The Warm Bath in Insomnia. - - - - - - 528

Dr. Gorgas' Experience with Borine. - - - - - 528

